# Supplementary material for: Notch Signaling Molecules as Prognostic Biomarkers for Acute Myeloid Leukemia
Source: Cancers (Basel). 2019 Dec 6;11(12):1958. doi: 10.3390/cancers11121958 (PMC6966525; doi:10.3390/cancers11121958)
Supplement: Supplementary file 1 [file cancers-11-01958-s001.docx]

**Table S1**

| Patient ID | Gender | Age | FAB | Risk Stratification |
| --- | --- | --- | --- | --- |
| Patient1 | M | 48 | M2 | Intermediate |
| Patient2 | F | 57 | M2 | Intermediate |
| Patient3 | F | 69 | M4 | Intermediate |
| Patient4 | F | 16 | M5 | Intermediate |
| Patient5 | F | 70 | M2 | Good |
| Patient6 | M | 64 | M4 | Adverse |
| Patient7 | M | 22 | M0/M1 | Intermediate |
| Patient8 | M | 35 | M0/M1 | Good |
| Patient9 | M | 62 | M1 | Good |
| Patient10 | M | 52 | M4eo | Good |
| Patient11 | M | 64 | M3 | Good |
| Patient12 | M | 34 | M2 | Adverse |
| Patient13 | F | 68 | M0/M1 | Intermediate |
| Patient14 | F | 27 | M3 | Good |
| Patient15 | F | 72 | sec MDS | Intermediate |
| Patient16 | F | 53 | M2 | Adverse |
| Patient17 | M | 61 | M1 | Adverse |
| Patient18 | F | 51 | sec MDS | Good |
| Patient19 | M | 70 | M4 | Good |
| Patient20 | F | 58 | M4 | Good |
| Patient21 | M | 27 | M2 | Good |
| Patient22 | M | 63 | M2 | Adverse |
| Patient23 | M | 34 | M4 | Adverse |
| Patient24 | F | 48 | sec MDS | Adverse |
| Patient25 | M | 67 | nas | Adverse |
| Patient26 | F | 62 | M4 | Good |
| Patient27 | M | 42 | M4 | Good |
| Patient28 | M | 60 | M2/M4 | Intermediate |
| Patient29 | M | 72 | M4 | Adverse |
| Patient30 | M | 35 | M2 | Good |
| Patient31 | M | 54 | M5 | Adverse |
| Patient32 | F | 68 | M4 | Good |
| Patient33 | F | 45 | M5 | Intermediate |
| Patient34 | M | 56 | M5 | Intermediate |
| Patient35 | F | 51 | M5b | Intermediate |
| Patient36 | M | 54 | M0/M1 | Adverse |
| Patient37 | F | 61 | M0/M1 | Intermediate |
| Patient38 | M | 19 | M4 eo | Good |
| Patient39 | M | 19 | M1 | Adverse |
| Patient40 | M | 66 | M0 | Adverse |
| Patient41 | M | 60 | M4/M5 | Intermediate |
| Patient42 | F | 67 | M4 | Adverse |
| Patient43 | M | 68 | M0 | Intermediate |
| Patient44 | F | 44 | M4 | Good |
| Patient45 | M | 65 | M1 | Good |
| Patient46 | M | 67 | M4 | Good |
| Patient47 | F | 69 | M2 | Intermediate |
| Patient48 | M | 59 | sec MDS | Adverse |
| Patient49 | F | 60 | M6/M7 | Adverse |
| Patient50 | M | 27 | M0 | Adverse |
| Patient51 | M | 40 | M2 | Good |
| Patient52 | F | 65 | M1 | Good |
| Patient53 | F | 40 | M2 | Good |
| Patient54 | M | 74 | sec MDS | N.V. |
| Patient55 | M | 38 | M2 | Intermediate |
| Patient56 | M | 57 | M4 | Adverse |
| Patient57 | F | 32 | M3 | Good |
| Patient58 | M | 44 | M0 | Intermediate |

**Table S1**: List of patients used for statistical analyses
